# Regional Requirements Influence Adoption of Exertional Heat Illness Preparedness Strategies in United States High Schools

**DOI:** 10.3390/medicina56100488

**Published:** 2020-09-23

**Authors:** Samantha E. Scarneo-Miller, Benjamin Saltzman, William M. Adams, Douglas J. Casa

**Affiliations:** 1 Division of Athletic Training, School of Medicine, West Virginia University, Morgantown, WV 26508, USA; 2 Korey Stringer Institute, Department of Kinesiology, University of Connecticut, Storrs, CT 06269, USA; Benjamin.saltzman@uconn.edu (B.S.); douglas.casa@uconn.edu (D.J.C.); 3 Heat, Environment and Thermal Stress Lab, Department of Kinesiology, University of North Carolina at Greensboro, Greensboro, NC 27412, USA; wmadams@uncg.edu

**Keywords:** best practice, adoption, policy and procedure, exertional heat stroke, preparation, regional differences, heat

## Abstract

*Background and objectives*: Exertional heat stroke (EHS) continues to be a prevalent health issue affecting all athletes, including our pediatric populations. The purpose of this study was to evaluate the effect of a state policy requirement for EHS prevention and treatment on local high school policy adoption in the United States (US). *Materials and Methods*: Athletic trainers (ATs) from high schools across the US participated in an online survey (*n* = 365). This survey inquired about their compliance with nine components of an EHS policy which was then compared to their state requirements for the policies. Evaluation of the number of components adopted between states with a requirement versus states without a requirement was conducted with a Wilcoxon Sign Rank test. Finally, an ordinal logistic regression with proportional odds ratios (OR) with 95% confidence intervals (CI) were run to determine the effect of a state requirement and regional differences on the number of components adopted. *Results*: ATs working in states with a requirement reported adoption of more components in their heat modification policy compared to states that did not require schools to develop a heat modification policy (with requirement mean = 5.34 ± 3.68, median = 7.0; without requirement mean = 4.23 ± 3.59, median = 5.0; *Z* = −14.88, *p* < 0.001). ATs working in region 3 (e.g., hotter regions) reported adopting more components than those in region 1 (e.g., cooler regions) (OR = 2.25, 95% CI: 1.215–4.201, *p* = 0.010). *Conclusions*: Our results demonstrate a positive association between state policy requirements and subsequently increased local policy adoption for EHS policies. Additionally, the results demonstrate that regional differences exist, calling for the need for reducing disparities across the US. These findings may imply that policy adoption is a multifactorial process; furthermore, additional regional specific investigations must be conducted to determine the true determinants of high school policy adoption rates for EHS policies.

## 1. Introduction

As exertional heat stroke (EHS) is one of the top three causes of sport-related death, it is imperative to implement strategies to prevent and treat this catastrophic injury [1]. The “National Athletic Trainers’ Association Position Statement: Exertional Heat Illness” (NATA-PS) sets forth guidelines supporting the assertion that the occurrence of EHS can be reduced and death can be prevented when proper strategies are in place [2]. Mainly, monitoring environment conditions can reduce the likelihood of EHS occurring (e.g., prevention) [2,3,4,5,6,7,8,9] whilst proper treatment using cold water immersion (CWI) initiated rapidly following an EHS diagnosis appears to eliminate the chance of death (e.g., treatment) [2,8,10,11]. While a number of modifiable and unmodifiable risk factors contribute to the onset of EHS, careful consideration, development, and implementation of various mitigation strategies has been evidenced to be successful in attenuating EHS risk [6,7,12].

Environmental factors (e.g., extreme heat and/or humidity) increase the risk of EHS as the external heat load impedes dissipation of body heat, especially during exercise. Environmental heat has long been considered a risk factor for EHS and is consistently cited within scientific and medical literature as a factor to address when developing heat mitigation strategies [2,3,4,5,7,8,10,13,14]. Recent evidence shows that EHS is more likely to occur when environmental conditions exceed normative data based on geographical location, thus prompting the need for region-specific activity modifications [3,4]. Current best practice recommendations advocate for the use of wet bulb globe temperature (WBGT) as the meteorological index that is most appropriate for quantifying environmental heat stress [2,8,13]. Furthermore, developing a series of graded activity modifications that are developed based on region specific meteorological and EHS epidemiologic data permit an evidence-based approach to risk mitigation.

Though activity modifications can reduce the likelihood of EHS occurring, it cannot eliminate the risk altogether. Therefore, there is a pressing need to ensure proper treatment strategies for those suffering from EHS. EHS is 100% survivable when proper treatment via CWI is promptly initiated [11]. It is imperative to cool the body as quickly as possible within 30 min of collapse and as such, cooling the body to under 38.9 °C (102°F) before transport to the hospital is crucial for a positive patient outcome. This mantra is often called “cool first, transport second”, indicating the need to cool the patient on-site, and then transfer to emergency medical services for transport to the nearest emergency department [15].

One approach to ensuring these best practice strategies for preventing and treating EHS are in place among high school athletics is the development, implementation, and adoption of policies. However, at the local high school level in the United States (US), several studies have identified a lack of overall compliance with safety standards related to exertional heat illness (EHI) prevention and treatment such as heat acclimatization and emergency action plans [16,17,18,19,20,21]. Conversely, when a policy is in place, it has been shown to be effective in improving patient outcomes following a catastrophic injury [22,23,24]. In the US, each state can set forth its own set of policy requirements for local high schools to follow. State policies have been shown to be effective at reducing EHS risk and has been identified as a facilitator for local emergency policy adoption [12,25,26,27]. Despite this, Adams et al. [28,29] found that very few states require their high school athletics programs to follow best practice recommendations with regards the prevention and treatment of EHS. The impact that state mandates have on local policy adoption for environmental monitoring and CWI remains unknown.

Given the severity of EHS amongst high school athletes in the US along with the continued change in the climate, it is of the upmost importance to better understand and enhance the standards of care and safety for these at-risk individuals. Ensuring policies and procedures for the prevention and treatment of EHS are adopted and implemented can reduce catastrophic outcomes from EHS. While research has shown state-level policies to be effective at reducing EHS risk, there is a paucity of evidence as to the effect of a state-level policy on the adoption of environmental monitoring and EHS treatment strategies. Therefore, the purpose of this study was to evaluate the effect of a state policy requirement for modification of activity in the heat along with other EHS treatment components, as defined in the NATA-PS, on local high school policy adoption [2].

**Hypothesis 1.**  *The prevalence of athletic trainers (ATs) reporting compliance with the environmental monitoring and EHS treatment strategies will be highest in states that require the adoption of the individual components outlined in the NATA-PS on EHS.*

**Hypothesis 2.**  *ATs working in states that require schools to develop a heat modification policy will adopt more components from the NATA-PS compared to ATs working in states that do not require schools to develop a heat modification policy.*

**Hypothesis 3.**  *ATs working in hotter climates with state requirements will adopt more components of the NATA-PS compared to those working in cooler regions.*

**Hypothesis 4.**  *ATs who report knowing their state has a policy requirement will be more likely to report adopting a policy.*

## 2. Materials and Methods

This study utilized a cross-sectional survey research design and an observational research design. This study was classified as exempt by the University of Connecticut Institutional Review Board.

### 2.1. Study Recruitment

The participants invited to partake in the survey included athletic trainers (ATs) employed in US high schools in the fall of 2018. ATs are healthcare professionals with training on the prevention, recognition, and care of sport-related injuries. ATs for this study were identified using the Athletic Training Locations and Services (ATLAS) Project [30]. Only participants from this source that consented to email contact for research purposes were contacted (*n* = 3119). Non-respondents received two follow-up emails, two weeks apart after the initial request was sent. Of the 3119 that were invited to take the survey, 439 surveys were started, and 409 were completed, yielding a valid response rate of 13.11%. Surveys were classified as complete if 80% or more of questions were answered.

### 2.2. Survey Instrument

The survey was developed based on the NATA-PS (Appendix A) [2]. The purpose of the survey was to gather data regarding local policy adoption of policies and their components for EHS prevention and treatment. Participants were asked to self-report if they had policies for the following nine topics: heat modification, use of an on-site WBGT device to measure the environment, regional specific guidelines, four levels of activity modification, work-to-rest ratios, use of shaded areas for rest breaks, modification of equipment, CWI tubs on-site for the treatment of EHS, and ensuring the patient is cooled below 38.8 °C (102 °F) before transport to the hospital.

### 2.3. Ascertainment of Data Related to State Requirements for Policy Implementation

A study performed by Adams et al. [28] sought to evaluate the ways in which sudden death and catastrophic injury prevention policies at the statewide level were being implemented at the local high school level. This was done by analyzing each state’s policy within multiple organizations, which included state high school athletic association, mandated state legislation, and the Department of Education. Policies were deemed relevant if they were required to be followed by all state high schools; therefore, any policy that was only recommended for implementation was not included in the evaluation. For use within this investigation, the data regarding environmental monitoring and CWI policy from the previously mentioned study will be utilized to assist in our evaluation (Table 1) [28]. With respect to the aforementioned study [28], only state-mandated policies that stem from either the state athletic association, state legislation, or the Department of Education will be included in our investigation. The Adams et al. [28] investigation was utilized in this study for the comparison of state-mandated policies on environmental monitoring and CWI policy adoption at local high schools.

### 2.4. Heat Safety Region

ATs were asked to report the zip code of their high school. Using this information, responses were categorized by the state the high school was in and into one of three heat safety regions based on previous research by Grundstein et al. [3]. Generally, region 1 encompasses a majority of the northern US, region 2 encompasses a majority of the middle of the US, and region 3 encompasses the Southeast and the southern portion of the US.

### 2.5. Statistical Analysis

Our study was looking to discover what impact state policy has on local policy adoption. The exposure variables were the presence of a state requirement. The main outcome (dependent variable) was the presence of a heat modification policy, component policies, and overall number of components adopted. Table 1 shows which states require policies for each of the environmental monitoring and EHS treatment guidelines. Statistical analyses for Hypothesis 1 were conducted in Microsoft Excel (Redmond, WA, USA). Statistical analyses for Hypothesis 2–4 were performed in SPSS version 26 (IBM Corp, Armonk, NY, USA). Statistical significance was determined a priori as *p* < 0.05.

#### 2.5.1. Covariates

The ATs in this sample provided demographic information including age, years of experience in the profession, years of experience in their high school, and school size by number of students during the 2018–2019 academic school year.

#### 2.5.2. Hypothesis 1

Similar to previous studies [18,31], we aimed to investigate the compliance with the environmental monitoring and EHS treatment components individually. Several best practice documents [2,8,13], including the NATA-PS, outline nine different components for environmental monitoring for the treatment of EHS [2]. As such, we aimed to evaluate if a state requirement facilitated improved compliance with the overall adoption of these components. Given that a state may require schools to develop a heat modification policy, but not the individual components of what should be included in that modification policy, we stratified the respondents into three groups: (1) respondents from states with both the heat modification requirement (HEAT ONLY); (2) respondents from states with both the heat modification policy and the specific components of that policy (BOTH); and (3) respondents in states without any requirements (WITHOUT). To evaluate if there was a difference in proportion between HEAT ONLY, BOTH, and WITHOUT, we conducted prevalence ratios (PRs). A PR with a 95% confidence interval (95% CI), excluding “1”, was considered statistically significant.

#### 2.5.3. Hypothesis 2

ATs were asked to identify the components of the heat policy in place at the high school in which they are employed. Medians and interquartile ranges are provided for the non-normally distributed data. To ascertain the effect a state policy has on the number of components adopted, we used a Wilcoxon signed-rank sum test to compare the distributions (0–9 components) between respondents from states with and without a requirement for the development of a heat modification policy.

#### 2.5.4. Hypothesis 3

A cumulative odds ordinal logistical regression with proportional odds was run to determine the effects of a state requirement, regional differences, age, number of students, years working in their school, and years in the profession overall on the number of environmental monitoring components adopted. For this analysis, we summed the number of components related to environmental monitoring from the survey (six policies) and compared it to whether or not the state required a heat modification policy and the different covariates.

#### 2.5.5. Hypothesis 4

We calculated chi-squared (χ^2^) tests of association with 95% CIs to evaluate the association between ATs identifying their state requires them to have a policy with (1) if the state did require the policy, (2) if their school had a policy, and (3) if they adopted all nine components vs. zero components. McNemar tests were calculated to evaluate the level of disagreement between ATs identifying they are required to have a policy and (1) if the state did require the policy, (2) if their school had a policy, and (3) if they adopted all nine components vs. zero components.

## 3. Results

### 3.1. Hypothesis 1: Component Analysis

Overall, when the state required schools to develop BOTH, ATs reported higher compliance with the individual components compared to HEAT ONLY and WITHOUT. Specifically, when states required both a heat modification policy and use of an on-site WBGT device in their policy, ATs were more likely to incorporate the on-site WBGT device in their policy compared to HEAT only and WITHOUT (Component #1, BOTH = 83.9%, HEAT ONLY = 66.9%, WITHOUT = 42.0%; BOTH vs. WITHOUT PR = 2.00, 95% CI: 1.60, 2.49; BOTH vs. HEAT ONLY PR = 1.25, 95% CI: 1.07, 1.57) (Table 2).

### 3.2. Hypothesis 2: Analysis of Number of Components Across Groups

**Figure 1:**
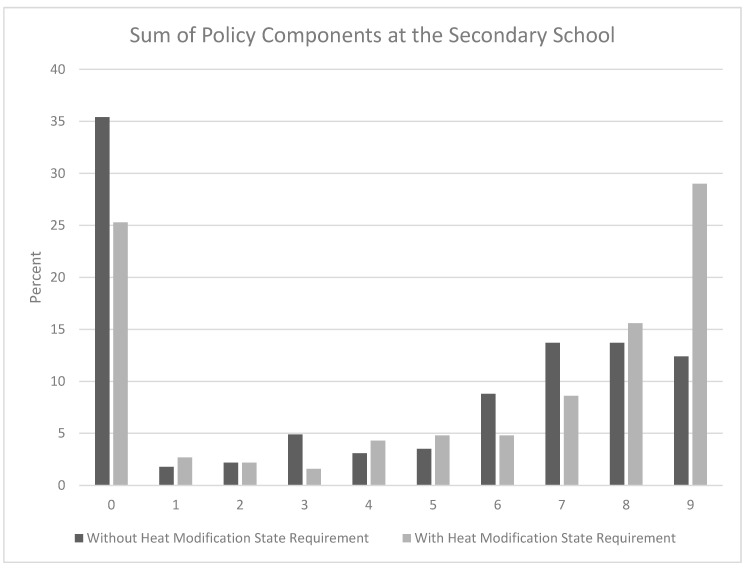
A Wilcoxon signed-rank test showed athletic trainers working in states that require schools to develop a heat modification policy complied with more NATA-PS components (median = 7.0, interquartile range (IQR) = 9.0) compared with athletic trainers working in states without requirements (median = 5.0, IQR = 8.0) (*Z* = −14.88, *p* < 0.001).

The Wilcoxon signed-rank test showed a statistical significant difference in overall adoption of the components in states that required a heat modification policy be developed and states without a policy (with requirement median = 7.0, interquartile range (IQR) = 9.0; without requirement median = 5.0, IQR = 8.0; *Z* = −14.88, *p* < 0.001) (Figure 1).

### 3.3. Hypothesis 3: Regional Comparison

A majority of the AT participants in this sample were from region 3 (region 1 = 21.2%, region 2 = 23.4%, region 3 = 55.4%). Despite this, a full likelihood ratio test identified proportional odds, comparing the fitted model to a model with varying location parameters (χ^2^(35) = 46.12, *p* = 0.099). The deviance goodness-of-fit test indicated that the model was a good fit to the observed data, χ^2^(1619) = 868.149, *p* = 1.00, but most cells were sparse with zero frequencies in 85.7% of the cells. However, the final model statistically significantly predicted the amount of components adopted over and above the intercept-only model (χ^2^(7) = 17.64, *p* = 0.014). ATs working in states with a heat modification policy requirement adopted more components of an environmental monitoring policy than those who worked in a state without a requirement (OR = 2.075, 95% CI: 0.744–3.017, χ^2^(1) = 10.269, *p* < 0.001). The regional differences had a statistically significant effect on the number of components (Wald χ^2^(2) = 14.427, *p* < 0.025). The odds of ATs working in region 3 adopting more components was 2.259 (95% CI = 1.215–4.201) times that of region 1 (Wald χ^2^(1) = 6.627, *p* = 0.010). There were no significant differences in odds between ATs working in region 3 and region 2 (χ^2^ = 2.304, *p* = 0.129) or region 2 vs. region 1 (χ^2^(1) = 1.283, *p* = 0.257). There was no difference between groups with any covariates (age (*p* = 0.859), number of students (*p* = 0.793), years in school (*p* = 0.932), or years in profession (*p* = 0.628).

### 3.4. Hypothesis 4: Knowledge of State Requirements

ATs were asked to identify if their state had a requirement for (1) an environmental monitoring policy and (2) a CWI policy for the treatment of EHS (Table 3). All but one analysis found significant association between the responses and significant disagreement between ATs identifying if a policy is required and if the state required it, if they had a policy, and if they adopted all components (*p* < 0.001–0.002, Table 3).

## 4. Discussion

Ensuring proper policies and procedures are in place for the monitoring of the environment as well as proper treatment strategies for those suffering from EHS is key to ensuring safe sport participation in the heat [2]. As such, the purpose of this study was to determine whether or not the presence of state policy mandates led to an increase in the rate at which local high school heat policies were adopted. Our findings suggest that when states require both a policy for heat modification and a specific component, schools are significantly more likely to adopt policies for both environmental monitoring and EHS treatment. Furthermore, the requirement of a state policy for heat modification appears to facilitate a greater number of components adopted in the high school’s heat policy. These associations are strengthened by the region of the country the AT is working in, in that those working in region 3 (e.g., the hotter regions) demonstrated a higher compliance with the components of an environmental policy compared to those working in regions 1 and 2. Finally, our findings show that ATs appear to know whether or not their state requires a policy but that it may not influence their school’s policy adoption.

When state officials and athletics administrators are considering their ability to affect high school student athlete health and safety, we would strongly encourage a state requirement for a heat modification policy that takes into account geographical meteorological conditions, work-to-rest ratios, types, timing and length of physical activity, and protective equipment considerations. Such a requirement appears to have a strong effect on both policy adoption and patient outcomes, as suggested in our results and previous research [12,18,25,26,27,31]. These results are paralleled by previous discoveries of Kerr et al. [18], who found that when states required heat acclimatization policies, high schools were more likely to be fully compliant with the NATA Inter-Association Task Force guidelines on heat acclimatization in states that mandated the guidelines. Similarly, ATs have reported a state requirement for emergency action plans to be a facilitator for the development of the policy [25,26]. Ultimately, while the purpose of this paper was not to evaluate the effect of a state policy on patient outcomes, we are encouraged to see that the presence of a state policy is influencing local policy.

As the climate continues to change, considerations must be made for the regional differences across state and local policies [3,4]. While the regional distribution of responses between ATs was uneven, the statistical tests (e.g., the proportional odds and deviance goodness-of-fit tests) support the use of the logistical modeling for the analyses of these data. The findings in this study show that ATs practicing in generally hotter climates (region 3) and in states with policy requirements were associated with adopting more components of an environmental monitoring policy. We can ascertain two main points from this finding: (1) states in region 3 should aim to develop policy requirements as soon as possible to enhance the local policy adoption standards for our most vulnerable athletes, and (2) the cooler climates (region 1 and 2) should aim to identify barriers to heat policy adoption. To the first point, these findings should not be surprising given that the ATs working at high schools in these hotter climates should be acutely aware of the risk imposed on their athletes. However, to the second point, ATs and states with high schools located in the cooler climates may not identify this risk to be as high, and thus are not preparing appropriately. We speculate that this may be due to the perception that the absolute maximum ambient temperatures observed in northern climates are not extreme enough to increase EHI/EHS risk. However, the relative changes in local temperatures (e.g., a maximum WBGT of 30°C in Boston on a given summer day when the normal average is 25°C) should be of greater concern when examining EHI/EHS risk profile. This is supported by prior work by Grundstein et al. [4], who found that 80% of EHS-related deaths occurring in northern climates happen when local environmental conditions exceeded their normal averages by greater than one standard deviation, compared to the EHS-related deaths occurring in southern climates, where 50% of those associated deaths occurred in climates at or below the local averages.

The final aim of this research study was to ascertain if ATs accurately identified if their state required policies for environmental monitoring and CWI. Most ATs appear to know whether or not their state had a policy; however, 31.2% of ATs were incorrect regarding environmental monitoring and 15.9% of ATs were incorrect regarding CWI in their identification of state required policy. Furthermore, knowledge of required policies did not appear to greatly influence adoption rates. This is an interesting finding given that previous research has suggested that ATs identify a state policy to be a facilitator for the development of their own policy—indicating that they would know if the state has a requirement, and it would facilitate greater adoption than if they did not have a requirement [25,26]. This signifies that policy adoption is likely influenced by many factors, not just one certain facilitator such as knowledge of a state policy or the presence of a state policy requirement. A multifaceted approach to policy adoption must be considered through using many different theories, frameworks, and models that combine previous literature together.

### Limitations and Future Directions

As a result of our analyses, we are hopeful in the main conclusions drawn from our study. Though this confidence exists, we cannot completely prove causality from our data, in that we cannot say with certainty that state policy is a direct facilitator of local policy adoption given that we had not controlled for all potential determinants of policy adoption. Some of the potential determinants we did not control for include socioeconomic status of the communities contact and the knowledge and behavior of the AT to adopt policies. Other limitations of our study include having a low response rate and subsequent small sample size. Future research may look to perform a similar study state-by-state to further regulate and analyze the previously mentioned potential regional barriers that our research could not control for. In addition, our research was evaluating policy that is intended to benefit patient outcomes, but these outcomes were not evaluated in our study. Future research may benefit from evaluating what the impact is from the presence of state policy on patient outcomes within states with, with some, or with no policy requirements for heat modification issues.

## 5. Conclusions

The results of this study indicate that policy and mandates appear to be positively associated with adoption of local high school policies related to EHS mitigation. The likelihood that a high school has a developed policy and outlined procedure for heat modification rises when a state mandates heat policy and its corresponding components (BOTH). These findings should inform state high school association officials and state legislators on the influence they have through the requirement of state policies on the development policies at the local high schools in an effort to best protect high school athletes. In the future, research must take a multifactorial approach that examines policy nationally, regionally, statewide, and locally in order to control for as many variables as possible to determine where the biggest influences of local policy adoption lie.

## Figures and Tables

**Table 1:** State requirements from Adams et al. [28].

POLICY	Number of States
State requires all schools to have a heat modification policy.	25 States
	AR, DE, GA, HI, IL, KS, KY, MD, ME, MI, MN, MS, MT, NC, NE, NJ, NM, NY, OH, OK, OR, SC, TN, VT, WI
**SPECIFIC COMPONENT OF HEAT POLICY**	
1. The heat policy is based on wet-bulb globe temperature (WBGT).	8 States
	GA, IL, MA, MN, NC, NJ, SC, VT
2. The environmental conditions guidelines are based on epidemiological data specific to that state/region.	10 States
	GA, IL, GA, ME, MN, NC, NJ, NY, SC, VT
3. The heat policy has at minimum four levels of modification, including the modification of practice time.	20 States
	DE, GA, HI, IL, KS, KY, MA, MI, MN, NC, NE, NJ, NM, NY, OH, OR, SC, TN, VT, WI
4. Policy includes modification of equipment.	21 States
	DE, GA, HI, IL, KS, KY, MA, ME, MI, MN, NC, NE, NJ, NY, OH, OK, OR, SC, TN, VT, WI
5. Policy includes modification of work:rest rations, including access to fluids.	20 States
	DE, GA, HI, IL, KS, KY, MA, MI, MN, NC, NE, NJ, NY, OH, OK OR, SC, TN, VT, WI
6. Policy mentions the use of shaded area for rest breaks.	9 States
	FL, GA, IL, KS, MA, NE, NJ, SC, VT
7. Cold water immersion tubs for on-site cooling for all warm weather practices.	10 States
	AR, GA, HI, ID, KY, MS, NC, NJ, UT, VT
8. If exertional heat stroke is suspected, on-site cooling using cold water immersion before transport to the hospital.	6 States
	AR, HI, NC, NJ, UT, VT

**Table 2:** Proportion of athletic trainers reporting compliance with a specific component of “National Athletic Trainers’ Association Position Statement: Exertional Heat Illness” (NATA-PS), by whether a state requires specific components of the heat policy or not.

Specific Component of Heat	Athletic Trainer Responses			Prevalence Ratio (95% CI)		
	States with Both Heat Policy and Component Mandates (BOTH)	States with Heat Mandate Only (Heat Only)	States with-out Heat and Component Mandates (WITHOUT)	With Both vs. Without	With Both vs. Heat Mandate Only	Heat Only vs. Without
1. The heat policy is based on WBGT.	52/62	91/136	60/143	2.00 *	1.25 *	1.59 *
	83.9%	66.9%	42.0%	(1.60–2.49)	(1.07–1.57)	(1.27–2.00)
2. The environmental condition guidelines are based on epidemiological data specific to that state/region.	58/66	110/133	119/144	1.06	1.06	1.00
	87.9%	82.7%	82.6%	(0.95–1.20)	(0.94–1.20)	(0.90–1.11)
3. The heat policy has a minimum of four levels of modification, including the modification of practice time.	100/116	115/136	106/144	1.17 *	1.02	1.15 *
	86.2%	84.6%	73.6%	(1.04–1.32)	(0.92–1.13)	(1.02–1.30)
4. The policy includes the modification of work:rest ratios, including unrestricted access to fluids.	102/120	114/136	104/144	1.18 *	1.01	1.16 *
	85.0%	84.6%	72.2%	(1.04–1.34)	(0.91–1.13)	(1.02–1.32)
5. The policy includes the modification of equipment.	103/119	115/135	119/1144	1.05	1.02	1.03
	86.6%	85.2%	82.6%	(0.94–1.16)	(0.92–1.12)	(0.93–1.14)
6. The policy mentions the use of a shaded area for rest breaks.	32/41	106/134	102/144	1.10	0.99	1.12
	78.0%	79.1%	70.8%	(0.91–1.35)	(0.82–1.19)	(0.97–1.28)
7. Cold water immersion tubs for on-site cooling for all warm weather practices	43/51	91/136	87/144	1.40 *	1.26 *	1.11
	84.3%	66.9%	60.4%	(1.17–1.67)	(1.07–1.49)	(0.93–1.32)
8. If exertional heat stroke suspected, on-site cooling using cold water immersion before transport to the hospital	34/41	104/136	110/155	1.09	1.08	1.00
	82.9%	76.5%	76.4%	(0.92–1.28)	(0.92–1.28)	(0.88–1.44)

* Denotes a statistically significant prevalence ratio (PR).

**Table 3:** Two-by-two contingency tables of athletic trainers (ATs) identifying if their state mandates them to have a policy compared to the state requiring it, if their school has the policy, and if they adopt all nine components.

		*n* (%)					
		Does the State Require This?		Does Your School Have Policies and Procedures on Exertional Heat Illness (Prevention and Treatment)?		Does Your School Adopt All Nine Components of an Exertional Heat Illness Policy?	
		No	Yes	No	Yes	No	Yes
Does your state mandate schools to have…							
An environmental monitoring policy?	No	179 (43.9)	81 (19.9)	100 (24.1)	167 (40.2)	234 (56.4)	99 (23.9)
	Yes	46 (11.3)	102 (25.0) ^a^	30 (7.2)	118 (28.4) ^b^	33 (8.0)	49 (11.8) ^c^
A policy for cold water immersion treatment of exertional heat stroke?	No	301(73.8)	32 (7.8)	109 (26.3)	231 (55.7)	284 (68.4)	56 (13.5)
	Yes	33 (8.1)	42 (10.3) ^d^	21 (5.1)	54 (13.0) ^e^	49 (11.8)	26 (6.3) ^f^

^a^ Significant association between ATs who identify that they are required to have an environmental policy and the state requiring the policy, χ^2^(1) = 54.38, *p* < 0.001, McNemar test, *p* < 0.002. ^b^ Significant association between ATs who identify that they are required to have an environmental policy and their school adopting a policy, χ^2^(1) = 13.06, *p* < 0.001, McNemar test, *p* < 0.001. ^c^ Significant association between ATs who identify that they are required to have an environmental policy and their school adopting all nine components of a policy, χ^2^(1) = 25.85, *p* < 0.001, McNemar test, *p* < 0.001. ^d^ Significant association between ATs who identify that they are required to have a cold water immersion (CWI) policy and the state requiring the policy, χ^2^(1) = 88.724, *p* < 0.001, McNemar test, *p* < 0.001. ^e^ Significant disagreement between ATs who identify that they are required to have a CWI policy and having an exertional heat illness (EHI) policy (McNemar test, *p* < 0.001); however, there was no association between cells, χ^2^(1) = 0.471, *p* = 0.492. ^f^ Significant association between ATs who identify that they are required to have a CWI policy and adopting all nine components of a policy, χ^2^(1) = 12.83, *p* < 0.001; however, McNemar test does not show disagreement between cells, *p* < 0.558.

## Appendix A

1. **Do you currently work in a high school?**

○ Yes
○ No

2. **What is your current role or position at your high school?**

○ Principal/Headmaster
○ Athletic Director
○ Head Coach
○ Assistant Coach
○ Nurse
○ Athletic Trainer
○ Parent of a Student-Athlete
○ Student-Athlete

3. **Age: ____________________________**
4. **How many students are enrolled at your high school? ________________________**
5. **How many years have you worked in your profession?**

○ Less than 1 year
○ 1–5 years
○ 6–10 years
○ 11–15 years
○ 15 or more years

6. **How many years have you served in your role at your school?**

○ Less than 1 year
○ 1–5 years
○ 6–10 years
○ 11–15 years
○ 15 or more years

7. **For each component, please select the category that best describes your high school’s current practice.**

**My high school’s written policy....**
**[participants answered yes or no]**

(a) Is based on environmental conditions measured by an on-site wet-bulb globe thermometer
(b) Is based on environmental conditions that are specific to my region of the country (regionally specific)
(c) Includes a minimum of four levels of modification, including the modification of practice time based on environmental conditions
(d) Includes modification of work:rest ratios based on environmental conditions
(e) Includes modification of protective equipment (if applicable to sport)
(f) Mentions the use of shaded areas for rest breaks
(g) Requires a cold water immersion tub to be on-site (within 5 min of each venue)
(h) Requires cooling on-site first, and then transport to the hospital (also known as “cool first, transport second”)

8. **Does your state mandate schools to have an environmental monitoring policy?**

○ Yes
○ No
○ I am not sure.

9. **Does your state mandate schools to have a policy for cold water immersion treatment of exertional heat stroke?**

○ Yes
○ No
○ I am not sure.
